# Mitogen-Induced B-Cell Proliferation Activates Chk2-Dependent G1/S Cell Cycle Arrest

**DOI:** 10.1371/journal.pone.0087299

**Published:** 2014-01-30

**Authors:** Pavel A. Nikitin, Alexander M. Price, Karyn McFadden, Christopher M. Yan, Micah A. Luftig

**Affiliations:** Department of Molecular Genetics and Microbiology, Center for Virology, Duke University School of Medicine, Durham, North Carolina, United States of America; University of Nebraska - Lincoln, United States of America

## Abstract

B-cell activation and proliferation can be induced by a variety of extracellular stimuli. The fate of an activated B cell following mitogen stimulation can be dictated by the strength or duration of the signal, the expression of downstream signaling components necessary to promote proliferation, and the cell intrinsic sensors and regulators of the proliferative program. Previously we have identified the DNA damage response (DDR) signaling pathway as a cell intrinsic sensor that is activated upon latent infection of primary human B cells by Epstein-Barr virus (EBV). Here we have assessed the role of the DDR as a limiting factor in the proliferative response to non-viral B-cell mitogens. We report that TLR9 activation through CpG-rich oligonucleotides induced B-cell hyper-proliferation and an ATM/Chk2 downstream signaling pathway. However, B-cell activation through the CD40 pathway coupled with interleukin-4 (IL-4) promoted proliferation less robustly and only a modest DDR. These two mitogens, but not EBV, modestly induced intrinsic apoptosis that was independent from the DDR. However, all three mitogens triggered a DDR-dependent G1/S phase cell cycle arrest preventing B-cell proliferation. The extent of G1/S arrest, as evidenced by release through Chk2 inhibition, correlated with B-cell proliferation rates. These findings have implications for the regulation of extra-follicular B-cell activation as it may pertain to the development of auto-immune diseases or lymphoma.

## Introduction

B lymphocytes respond to pathogens through a highly regulated process that includes a period of rapid proliferation concomitant with targeted DNA damage at the immunoglobulin (Ig) locus. This process is under tight spatial and temporal control achieved by extracellular signals sensed via B cell receptor (BCR), CD40 receptor, Toll-like receptors (TLR), B-cell activating factor (BAFF) receptor and cell intrinsic mechanisms (reviewed in [1]). Upon antigen engagement, B cells are induced to transition from quiescence to the G1 phase of the cell cycle. A second signal mediated by the interaction of the T cell expressing CD40 ligand (CD40L) with the CD40 receptor on B cells in conjunction with T-cell derived cytokines is required for their survival and proliferation [2]. Alternatively, ligands for TLRs, including TLR9, can directly provide signals necessary to initiate B-cell proliferation [1]. Epstein-Barr virus (EBV) infects resting B cells and promotes proliferation by mimicking T-cell derived signals [3], [4].

Activated B cells in lymphoid tissue form germinal centers (GCs) where they undergo rapid proliferation in response to antigen and T-cell derived cues. Within GCs, B cells undergo affinity maturation through somatic hyper-mutation of their Ig genes and class switch recombination (CSR), both mediated by activation-induced cytidine deaminase (AID) [5]–[8]. These AID-mediated functions result in B cells individually expressing unique and diverse antibodies that can interact with the antigen with high affinity and express different effector functions through the Fc portion of the molecule. These processes involve the formation of AID-induced double stranded breaks (DSBs) at the Ig loci and generally occur during the G1 phase of the cell cycle. Off target AID double stranded breaks do not appear to occur frequently in normal dividing cells although they can be induced with genetic manipulation [9]. CSR occurs during B-cell proliferation and has been directly linked to the number of divisions that the B cell goes through [10]. B cells may also incur DSBs due to replicative stress induced by the rapid pace of proliferation in GCs, which could result in the depletion of nucleotide pools leading to replication fork arrest or hyper-origin firing and subsequent replication fork collision. However, within the GC, these activities are attenuated by the transcriptional repression of the ssDNA damage sensor, ATR, by Bcl-6 [11]. Uncontrolled activation and proliferation of B lymphocytes outside of the GC environment, e.g. extra-follicularly, could result in DNA damage response (DDR) activation with untoward consequences on cell proliferation or survival. In fact, uncontrolled activation coupled with cellular proliferation have been linked to pathologies such as autoimmune disorders [12] and lymphomas [13].

The DNA damage response (DDR) has been recognized as one of the major innate growth suppressive mechanisms activated in hyper-proliferating cells [14]–[17]. Multiple molecular sources have been implicated in hyper-proliferation induced DDR including replication fork collapse, telomere exposure, and the accumulation of reactive oxygen species [16]. In nearly all cases, these events lead to activation of PI3 kinase-like kinases including ataxia-telangiectasia mutated (ATM). ATM then activates a number of downstream effectors including the checkpoint kinase Chk2. In turn, activated ATM and Chk2 induce p53-mediated cell cycle arrest, or with extensive irreparable damage apoptosis or senescence [14],[17]–[19].

Work from a number of groups suggests that there is a role of ATM and Chk2 in suppressing lymphomagenesis [20]–[23]. Our group identified the ATM/Chk2-dependent DDR as a suppressor of Epstein-Barr virus (EBV) mediated transformation of primary human B cells *in vitro* [24]. Further, we demonstrated that the induction of the DDR occurred independently from the replication of viral DNA and was attributed to a transient period of hyper-proliferation during the first 3–4 division of human B cells. However, whether non-viral activators of B-cell proliferation induce a DDR and what the downstream consequences of DDR activation might be in mitogen-stimulated B cells remains unknown. Here we study the role of the ATM/Chk2 signaling pathway in response to B-cell proliferation following activation of T-cell independent TLR signaling or T-cell mimicking CD40 signaling coupled to IL-4.

## Results

### Mitogen Stimulation of Primary B Cells Results in an Initial Period of Robust Proliferation

We first determined the proliferative kinetics of human B cells in response to diverse mitogens. Human PBMCs were labeled with the fluorescent tracking dye Cell Trace Violet and infected with the B95-8 strain of EBV or stimulated with two distinct B-cell mitogens. To simulate signaling through the TLR pathway we treated PBMCs with the TLR9 ligand CpG (ODN 2006) that mimics common patterns on bacterial DNA (termed “CpG”) [25], [26]. We mimicked T-cell dependent signaling by activating the CD40 receptor in combination with the cytokine IL-4. B-cell proliferation was monitored by flow cytometric analysis of CD19+/Violet^lo^ cells and the mean division number (MDN) was calculated for each time point as previously described [24], [27].

**Figure 1:**
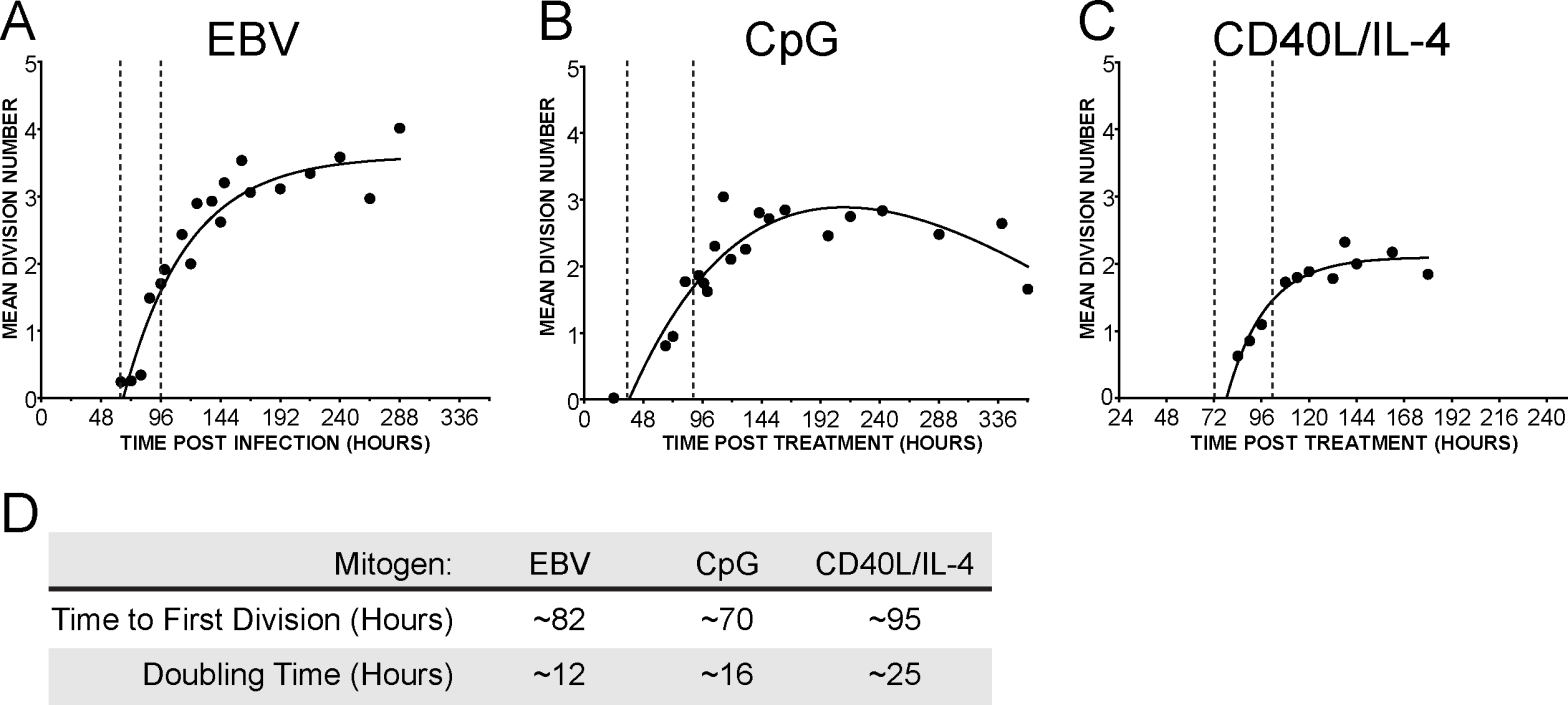
Multiple B-cell mitogens induce an initial burst of hyper-proliferation. Kinetics of B cell proliferation in response to (A) Epstein-Barr virus infection, (B) constant stimulation with TLR9 ligand CpG (2.5 µg/mL), or (C) CD40L (5 ng/mL) coupled with interleukin-4 (20 pg/mL) treatment. The mean division number based on precursor cohort analysis [42] and plotted over time post infection or treatment is shown. Vertical dashed lines indicate the period of hyper proliferation. (D) The mean division number and time to first division was calculated as in [24], [27] for the period of hyper-proliferation.

B-cell activation by all three stimuli resulted in an initial burst of proliferation followed by a plateau phase characterized by a substantially slower proliferation rate (Figure 1A–C). As has been previously observed, CpG and CD40L/IL-4 treatment ultimately led to a loss of proliferation and cell death while EBV infected cells continued to proliferate as immortalized lymphoblastoid cell lines (LCLs) [27], [28]. Both the rate of proliferation and time to first division varied with the differed stimuli (Figure 1D). EBV-infected cells began dividing three days after infection and doubled approximately every 12 hours for the first several days before proliferation slowed to the rate observed in immortalized B cells (Figure1A and [24]). CpG-stimulated B cells had the shortest time to first division and an initial period of hyper-proliferation with a doubling time of 16 hours that ultimately waned (Figures 1B and 1D). In contrast, cells stimulated to divide through the CD40 receptor divided at a rate of approximately once every 25 hours (Figures 1C and 1D). This rate is slower than the burst observed by EBV infection or CpG treatment, but the pattern of a short period of rapid proliferation followed by a plateau phase remained constant.

### Mitogen Stimulation Activates the ATM Signaling Pathway in Proliferating B Cells

**Figure 2:**
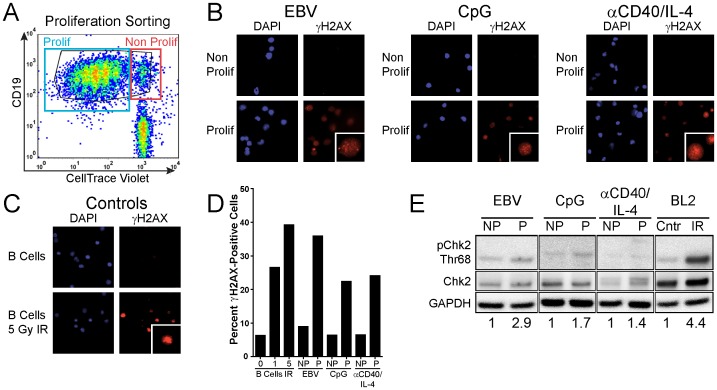
B-cell mitogens activate the ATM signaling pathway in proliferating cells. (A) Representative FACS plot of stimulated PBMCs showing the distribution of Cell Trace Violet stained cells versus CD19 staining for B cells. The indicated proliferating and non-proliferating populations were isolated by FACS sorting for subsequent analysis. (B) Immunofluorescence microscopy for γ-H2AX (red) of sorted non-proliferating (Non Prolif) or proliferating (Prolif) B cells. The DAPI stains for DNA are shown. (C) Untreated and 5 Gy γ-irradiated controls assayed as described in (B). (D) The average percentage of cells with γ-H2AX intensity >5X over background are plotted for three normal donors. Samples include B cells, untreated (0), 1 Gy, or 5 Gy γ-irradiation, sorted non proliferating (NP) and proliferating (P) cells for EBV, CpG, or αCD40/IL-4 treatments. Quantification is detailed in Figure S1. (E) Western blot analysis of Chk2, Chk2 phosphorylated on Thr68 as well as the control GAPDH. Untreated (Cntr) and 5 Gy irradiated (IR) Burkitt’s lymphoma cell line 2 (BL2) are included as a positive control. Numbers indicate the normalized densitometry value for phospho-Chk2 Thr 68.

We previously demonstrated that the transient period of hyper-proliferation in EBV-infected B cells corresponds with the induction of hallmarks of the DDR [24]. To determine if B cells stimulated with other mitogens also induce a DDR, we FACS sorted mitogen stimulated B cells to separate cycling (“proliferating”) and quiescent (“non-proliferating”) cells (Figure 2A) and probed for characteristic markers of the DDR including the presence of γ-H2AX and signaling through the ATM pathway. Elevated levels of γ-H2AX were detected in the proliferating cells following EBV infection, CpG stimulation, and CD40L/IL-4 treatment relative to the non-proliferating population (Figures 2B–2D and S1). The highest levels of γ-H2AX were observed in the EBV-infected proliferating B cells, with levels similar to what was observed in B cells subjected to 1 to 5 Gy of gamma irradiation consistent with previous observations [24]. We also observed an increase in the phosphorylation of the ATM effector kinase, Chk2, on Thr-68 in proliferating cells relative to the non-proliferating population (Figure 2E). This was most clearly evident in the rapidly proliferating EBV-infected and CpG-treated B cells, whereas cells stimulated through the CD40 pathway displayed only a modest increase in Chk2 phosphorylation.

### EBV and Mitogen-induced B-cell Proliferation is Suppressed by Chk2

**Figure 3:**
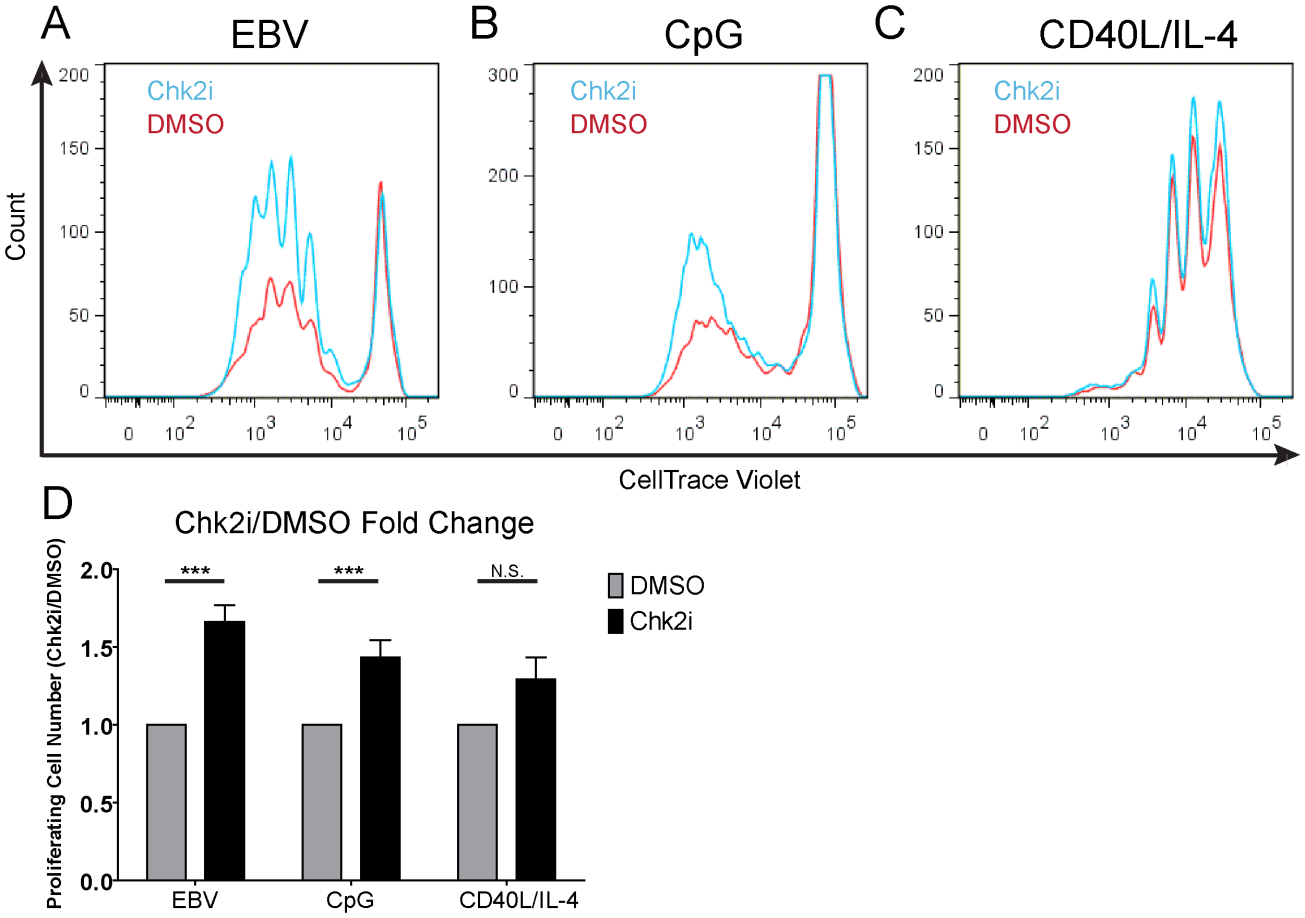
Chk2 inhibition increases proliferation of human B cells. FACS profile of (A) EBV infected (B) CpG or (C) CD40L/IL-4 treated CD19+ cells at the day of peak proliferation post infection/stimulation in the presence of DMSO (red) or 5 µM Chk2i (blue). For EBV this was day 8 post infection, CpG was day 7 post stimulation, and CD40L/IL-4 was day 6 post stimulation. (D) Relative number of proliferating CD19+ cells were gated and counted over internal beads control and normalized to DMSO-treated sample on the days described in (A–C). Experiment was repeated on PBMC from 3–6 normal donors. Error bars are SEM. Significance of Chk2i treatment compared to the DMSO control was calculated via Student’s *t* test; ***, *P*-value <0.01; N.S., not significant. CD40L/IL-4 treatment trended towards significance with a *P*-value of 0.078.

To determine if the DDR alters the proliferation of mitogen-treated cells, we stimulated or EBV-infected B cells in the context of PBMCs derived from at least three independent normal human donors in the presence of a small molecule inhibitor that blocks the kinase activity of Chk2 [29]. B-cell proliferation was monitored by CD19 staining and dilution of Cell Trace Violet proliferation tracking dye. Consistent with our previous observations [24], there was a significant increase in the number of proliferating EBV-infected B cells in the presence of the Chk2 inhibitor (Chk2i) relative to DMSO control at 8 days post infection (Figures 3A and 3D). While Chk2 inhibition increased EBV-induced B-cell proliferation by ∼50% at this early time, the long-term consequence of this effect is a nearly 10-fold increase in EBV transformation efficiency [24]. Moreover, this phenotype was independent of EBV multiplicity of infection (data not shown). Chk2 inhibition promoted a similarly significant increase in the number of proliferating CpG-treated B cells 7 days post stimulation (Figures 3B and 3D). Changing the dose of CpG used to stimulate peripheral blood B cells did not significantly alter the initial proliferative response (data not shown). Consistently, Chk2 inhibition increased CpG-induced B-cell proliferation from 25 nM to 100 nM (data not shown). Finally, stimulation through the CD40 receptor coupled with IL-4 resulted in a modest, though not statistically significant, increase in the number of proliferating B cells (Figures 3C and 3D). As expected, then, altering the strength of the CD40 signal did not impact the ability of Chk2 to impinge on proliferation. Therefore, the rate of B-cell hyper-proliferation correlated with the sensitivity of these cells to Chk2 inhibition: EBV>CpG>CD40+IL-4.

While Chk2 inhibition increased the number of proliferating B cells after stimulation with EBV and CpG, we did not observe a change in the time to first cell division in Chk2i-treated cells relative to DMSO (data not shown). Rather, inhibition of Chk2 increased the number of B cells entering subsequent divisions. These data suggest that a growth-suppressive mechanism is operative in hyper-proliferating B cells acutely following mitogen stimulation.

### B-cell Mitogens Induce Caspase 3/7-dependent Apoptosis Independent of Chk2

**Figure 4:**
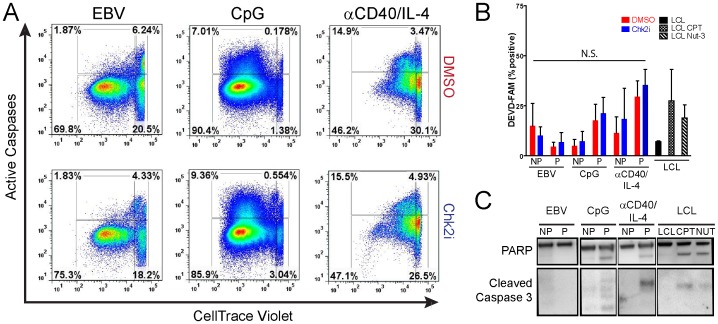
B-cell mitogens, but not EBV, induce caspase 3/7-dependent apoptosis. (A) Activated apoptosis in proliferating B cells treated with DMSO (top panels) or Chk2i (bottom panels) is measured with DEVD-FAM, a fluorescently labeled soluble caspase 3/7 inhibitor. FACS profiles represent CD19+ single cells. (B) Percent of active caspase-positive cells is plotted for non-proliferating (NP) or proliferating (P) EBV-infected, CpG treated, or αCD40/IL-4 treated CD19+ cells in the presence of DMSO (red) or Chk2i (blue). By comparing the Chk2i treated or DMSO treated conditions we found no statistically significant difference in the percentage of active caspase-positive cells by Student’s *t* test (*P*-value >0.05; N.S., Not Significant). Activation of caspases was compared to LCLs treated with 15 µM camptothecin (CPT) or 10 µM Nutlin-3 (Nut-3). (C) Cleavage of caspase targets (PARP and self-processing of caspase-3) revealed by western blot analysis of sorted non-proliferating (NP) or proliferating (P) EBV-infected or mitogen-stimulated B cells.

The DDR signaling pathway through ATM and Chk2 leads to both growth arrest and apoptosis [30]. We therefore first queried the role of Chk2 in promoting apoptosis in EBV-infected or mitogen-stimulated proliferating B cells. Cells stimulated with treatment by both CpG and αCD40/IL-4 induced apoptosis, revealed by increased signal from a fluorescently labeled caspase 3/7 substrate DEVD-FAM (Figures 4A and 4B) and by Western analysis of cleaved PARP and caspase 3 (Figure 4C). However, the level of apoptosis, as measured by caspase activation, was not altered by Chk2i treatment (compare Figure 4A top to bottom panels, or 4B). We did not observe many caspase-positive apoptotic cells in EBV-infected early proliferating B cells consistent with earlier reports (Figure 4A–4C and [28]). Therefore, we conclude that Chk2 inhibition does not relieve activation of caspase 3/7 as a means to increase the number of proliferating mitogen-stimulated B cells.

### Mitogen-induced Proliferation of Human B Cells Induces a Chk2-dependent G1/S Cell Cycle Arrest

**Figure 5:**
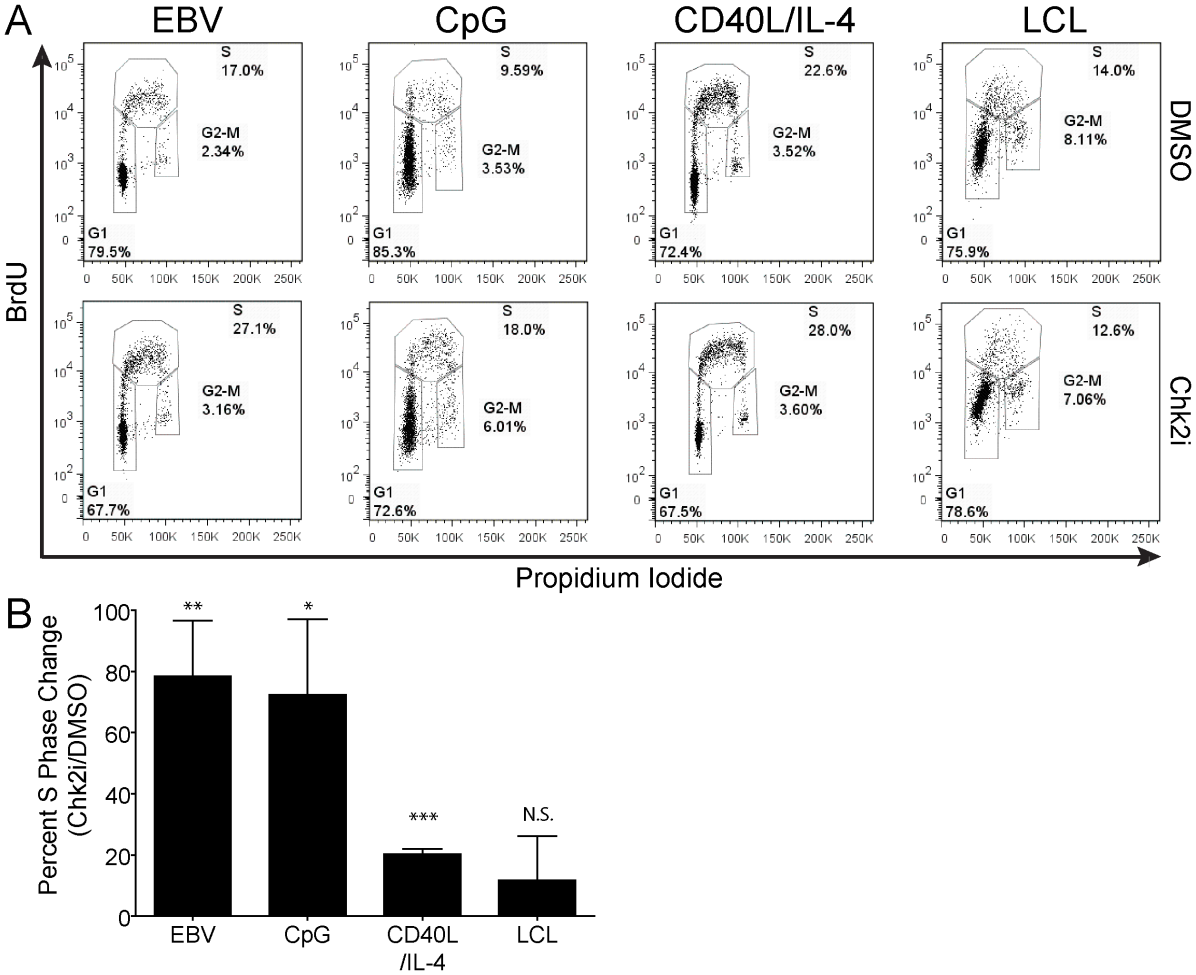
Mitogen stimulation or EBV infection of human B cells activates Chk2-dependent cell cycle arrest. (A) Cell cycle profile of EBV-infected or mitogen-induced proliferating CD19+9 B cells treated with DMSO or Chk2i measured by FACS. Cells were pulsed with 25 µM BrdU for 2 hours and subsequently stained with anti-BrdU antibody and propidium iodide (PI). G1, S, and G2/M stages of the cell cycle are indicated. (B) Percent change of cells (from (A)) in S-phase in Chk2 treated over DMSO-treated samples from four to seven normal donors. Error bars are SEM. Significance was calculated with a Student’s *t* test. N.S., not significant; *, *P*-value <0.05; **, *P*-value <0.01; ***, *P*-value <0.0001.

Activated Chk2 has been shown to induce cell cycle arrest through the phosphorylation of a number of downstream effectors including p53 and Cdc25 [31]. To determine if the DDR suppresses the number of mitogen-stimulated proliferating B cells through the establishment of a cell cycle checkpoint, we assayed the cell cycle profile of infected or stimulated, proliferating B cells following a 2 hour pulse with the thymidine analog BrdU. B cells infected with EBV or stimulated with CpG had approximately 70–80% more cells entering S phase in Chk2i-treated cells relative to DMSO treated controls (Figures 5A and 5B). In contrast, the slower proliferating CD40L/IL-4 treated cells had a modest, but significant, increase in cells entering S phase after inhibition of the Chk2 pathway (Figures 5A and 5B). We observed no difference in the number of Chk2i-treated LCLs entering S phase with up to six days continuous treatment. In addition, we did not observe a significant alteration in the numbers of cells present in G2/M phase in any condition tested. Therefore, a Chk2-dependent G1/S checkpoint limits EBV-infected and mitogen-driven B-cell growth in a manner correlated with the extent of hyper-proliferation.

## Discussion

In this study, we have identified a Chk2-mediated G1/S phase cell cycle checkpoint activated upon the initial proliferation of mitogen-stimulated B cells. Similar to EBV infection of primary human B cells, CpG stimulation of TLR9 drives a period of hyper-proliferation, while CD40 signaling coupled to IL-4 drives a transient period of proliferation with an attenuated rate through the first two cell divisions. Importantly, similar to EBV infection, TLR9 and CD40/IL-4 signaling activate an ATM signaling pathway during these early cell divisions. The activation of this DDR signaling pathway does not lead to B-cell apoptosis, but rather to a specific G1/S phase arrest that limits proliferation. These results suggest that the DNA damage response acts as an early barrier preventing aberrant proliferation of extra-follicular activated B cells.

### EBV-driven Hyper-proliferation Activates a G1/S Phase Cell Cycle Arrest, but not Apoptosis

EBV infection of primary human B-lymphocytes activates a growth-suppressive ATM/Chk2-dependent DDR [24]; however the precise nature of this growth suppression remained unknown. Here we have revealed that a Chk2-dependent G1/S cell cycle arrest suppresses the EBV-driven hyper-proliferation of human B cells. Notably, we did not detect active apoptosis in infected early proliferating cells, consistent with previous findings [28] and suggests that viral BCL2 homologs expressed during the early stage of latent infection may play a role in B-cell survival [32].

The DDR-dependent G1/S arrest was readily detected in early proliferating B cells but not in indefinitely transformed LCL, despite the fact that LCLs retained a low level of γ-H2AX positivity [24]. Recent reports demonstrated the formation of persistent heterochromatic foci with active DDR signaling in proliferating cells [33], [34]. Specifically, Rodier, *et al*. identified sites called DNA-SCARS (DNA segments with chromatin alterations reinforcing senescence) as foci with persistent ATM-downstream signaling in cells driven to senesce by oncogenic stress or exogenous stimuli [33]. Interestingly, such cells could proliferate in the presence of DNA-SCARS if the p53 or Rb tumor suppressor pathways were inhibited [33]. Intriguingly, the EBV EBNA-3C protein attenuates the DDR-mediated growth arrest in early infected cells [24] and induces epigenetic inactivation of p16 in transformed LCLs [35]–[37]. Therefore, it is reasonable to conclude that EBNA2-dependent transcription of host growth promoting genes, such as c-Myc, initiates oncogene-induced replicative stress and activation of ATM/Chk2-dependent G1/S growth arrest of the majority of infected proliferating cells, while later in infection EBNA2 driven EBNA3C attenuates the expression of oncogenes and inactivates p16 allowing subsequent cellular proliferation despite low, but persistent ATM signaling. A complementary role of EBNA-3C early in infection may be to attenuate p16 thereby preventing exacerbation of the DNA-SCARS phenotype [33].

### TLR9 Stimulation of Human B Cells Activates Two Independent Growth-suppressive Mechanisms

We have demonstrated a bi-phasic profile of human B-cell proliferation in response to TLR9 stimulation with CpG *in vitro*. Particularly, during the first three divisions proliferating B cells were free of apoptotic markers and displayed Chk2-dependent and reversible G1/S cell cycle arrest. However, divisions four and higher displayed apoptotic markers, including activated caspases 3 and 7 and cleaved PARP. Consistent with our findings, CpG-induced dividing murine BCL2 transgenic B cells remained alive while not dividing in late divisions [27]. Therefore, together with these studies by the Hodgkin group, our data suggest that the two factors regulating TLR9-induced proliferation of human B-cells *in vitro* [38] are, first, a DDR-dependent G1/S cell cycle arrest and, second, DDR-independent and proliferation-linked apoptosis.

### CD40 Signaling Coupled with Interleukin-4 Induces Modest Proliferation and G1/S Arrest

B cells stimulated to proliferate through the CD40 pathway and IL-4 exhibited a slower proliferative burst relative to CpG-stimulated or EBV-infected cells. While these cells did exhibit the formation of γ-H2AX foci, they displayed only modest activation of Chk2. Consistently, these cells were only minimally responsive to the inhibition of the Chk2 pathway as seen with the modest increase in the number of cells entering S phase as well as a minimal increase in the total number of proliferating B cells.

### Physiological Consequences

Rapid proliferation of B cells occurs in the dark zone of the germinal center. It might be expected that a growth-suppressive DDR is activated in this setting. However, elegant studies by Ari Melnick’s group suggest that the key GC transcription factor Bcl-6 directly represses expression of the DDR replicative stress sensing kinase ATR and prevents its expression in centroblasts [11]. As shown here, extra-follicular B-cell proliferation induced by T-cell independent signals such as CpG DNA or EBV infection can lead to the hyper-proliferation of B cells and activation of a growth suppressive DDR. In the absence of additional survival signals or factors maintaining prolonged proliferation, these cells will die or arrest. In the case of EBV infection, a switch in viral latency promoters enables long-term outgrowth through expression of additional latency proteins allowing attenuated proliferation and increased survival [24], [39]. CpG DNA stimulates a burst of B-cell proliferation that may work in concert with cytokines or, ultimately, cognate T-cell signals to promote survival and differentiation. In the absence of these proper controls or upon mutation of cell intrinsic regulators of survival (e.g. Bcl2 translocation [40] or MyD88 activating mutations found in many B-cell lymphomas [41]), the consequences of DDR activation and limiting extra-follicular proliferation can be the promotion of lymphomagenesis or increase survival of auto-reactive B cell clones leading to auto-immunity [42]. Future studies toggling DDR signaling *in vivo* during extra-follicular B-cell activation and EBV infection will be important to address such pathophysiological consequences.

## Materials and Methods

### Cells, Virus, and Mitogens

Buffy coats were obtained from normal donors through the Gulf Coast Regional Blood Center (Houston, TX) and peripheral blood mononuclear cells (PBMC) were isolated by Ficoll Histopaque-1077 gradient (Sigma #H8889). CD19+ B cells were either purified from PBMC using the BD iMag Negative Isolation Kit (BD #558007) or from buffy coats using the RosetteSep kit (Stem Cell, cat #15024). Purity was routinely greater than 90% as determined by flow cytometry. Chk2 was inhibited using the 2-arylbenzamidazole compound Chk2i II (EMD Millipore #220486) [29]. Primary human B cell infection with B95.8 strain of Epstein-Barr virus was performed as previously described [24]. Thioester stabilized TLR9 ligand CpG ODN 2006 oligonucleotide [25] was purchased from IDT and used at 2.5 µg/ml. mAb G28-5 that binds and activates human CD40 was prepared from a hybridoma cell line (ATCC HB-9110, kind gift of E. Kieff, Harvard Medical School) and used at the final concentration of 1 µg/ml. Human recombinant interleukin-4 (PeproTech #AF200-04) was used at 20 ng/mL. CD40 ligand was purchased from (R&D Systems #6420-CL) and used at 5 ng/ml in combination with an anti-HA peptide cross-linking antibody (R&D Systems #MAB060) at a concentration of 0.2 µg/µl. Experiments were performed using either soluble CD40L or the agonistic CD40 antibody G28-5 to activate the CD40 receptor together with IL-4 stimulation. Similar results were obtained with either stimulus.

### Antibodies

Primary antibodies to γ-H2AX, pATM Ser1981, and pChk2 Thr68 (Cell Signaling Technology #2577, #4526, and #2197 respectively) were used at 1∶1000 in Western protein assay and at 1∶50 in immunofluorescence microscopy. Alexa488 goat anti-mouse and Alexa 568 goat anti-rabbit were used as secondary antibodies (Molecular Probes #A11029 and #9654). Mouse anti-human CD19 antibody conjugated with APC (BD Bioscience #555415) was used as surface B cell marker in flow cytometry. Cleaved PARP and caspase 3 proteins were detected in Western blot assays using Roche #11835238001 and Cell Signaling Technology #9654, respectively.

### Immunofluorescent (IF) Microscopy

IF was performed as previously published [24]. 5×10^5^ CD19^+^ B cells in suspension were pelleted, washed in PBS, resuspended in 40 µl of PBS, spread on a microscope slide and dried at 37°C for 20 minutes then fixed in 4% paraformaldehyde in PBS for 15 minutes, permeablized in PBS containing 0.5% Tween-20 for 20 minutes and blocked in PBS with 0.2% Tween-20 containing 5% normal goat serum for 1 hour. Indirect immunofluorescence was performed as described in [24]. Slides were mounted in Vectashield containing DAPI (Vector Laboratories).

### Protein Expression Analysis

Cells were pelleted and washed in PBS, and then lysed in 0.1% triton-containing buffer. Protein lysates were separated using NuPage 4–12% gradient gels (LifeTechnology) and transferred to PVDF membrane (GE Healthcare). Membranes were blocked in 5%BSA in TBST and stained with primary antibody overnight at +4°C, followed by a wash and staining with secondary HRP-conjugated antibody for 40 min at room temperature.

### Flow Cytometry Analysis and Cell Sorting

CellTrace Violet (Invitrogen #C34557) stained PBMCs were induced to proliferate with mitogens or infected by EBV and incubated with 5 µM concentration of Chk2i or treated with an equivalent volume of DMSO. At different times post mitogen induction or viral infection, the PBMCs were stained with fluorescent antibodies. FACS analysis was performed at BD Canto II machine. Kinetics of B-cell proliferation was determined as previously described [24], [27].

### Apoptosis Assay

FACS-based detection of activated caspases 3/7 was performed on primary PBMC using Molecular Probe Vybrant FAM-DEVD (Cat #9654) assay used as directed by the manufacturer at days 4–6 post stimulation or infection.

### Cell Cycle Analysis

Proliferating cells were pulsed with 25 µM thymidine analog BrdU for 2 hours and then fixed with 1% paraformaldehyde for 30 min and permeabilized with 0.5% Triton in PBS for 15 min at +4°C. Permeabilized cells were thoroughly washed and treated with DNase I (Sigma #AMPD1) for 40 min at +37°C in a supplied buffer. Alternatively, cells were fixed with 70% ice-cold ethanol overnight followed by denaturation with 2 M HCl for 30 minutes with periodic agitation and neutralization with 0.1 M Sodium Tetraborate, pH 8.5. After that PBMC were washed, blocked with 1% goat serum and stained with anti-BrdU antibody (BD Biosciences #560209) at 4°C for 1–2 hrs then washed and stained with anti-mouse secondary fluorescent antibody. After that stained cells were incubated with 25 µM RNAse A and 50 µM propidium iodide.

### Ethics Statement

The studies reported in this manuscript used human peripheral blood cells from normal donors provided by the Gulf Coast Regional Blood Center (Houston, TX). These samples contained no HIPAA identifiers and as such are considered exempt from human subjects research approvals (Duke IRB exemption #Pro00006262).

## Supporting Information

Figure S1 Quantification of γ-H2AX Immuno-Fluorescence intensity. Intensity for γ-H2AX immune-fluorescence staining for three normal human donors shown in Figure 2B is displayed in column format. Samples include B cells, untreated (0), 1 Gy, or 5Gy γ-irradiation, sorted non proliferating (NP) and proliferating (P) cells for EBV, CpG, or αCD40/IL-4 treatments. Mean fluorescence is shown by red bars. Significance was calculated using the non-parametric Mann-Whitney *U* test. ***, *P*-value <0.001; N.S., not significant.
(TIF)
