# Supplementary figures and images for: Mitogen-Induced B-Cell Proliferation Activates Chk2-Dependent G1/S Cell Cycle Arrest
Source: PLoS One. 2014 Jan 30;9(1):e87299. doi: 10.1371/journal.pone.0087299 (PMC3907503; doi:10.1371/journal.pone.0087299)

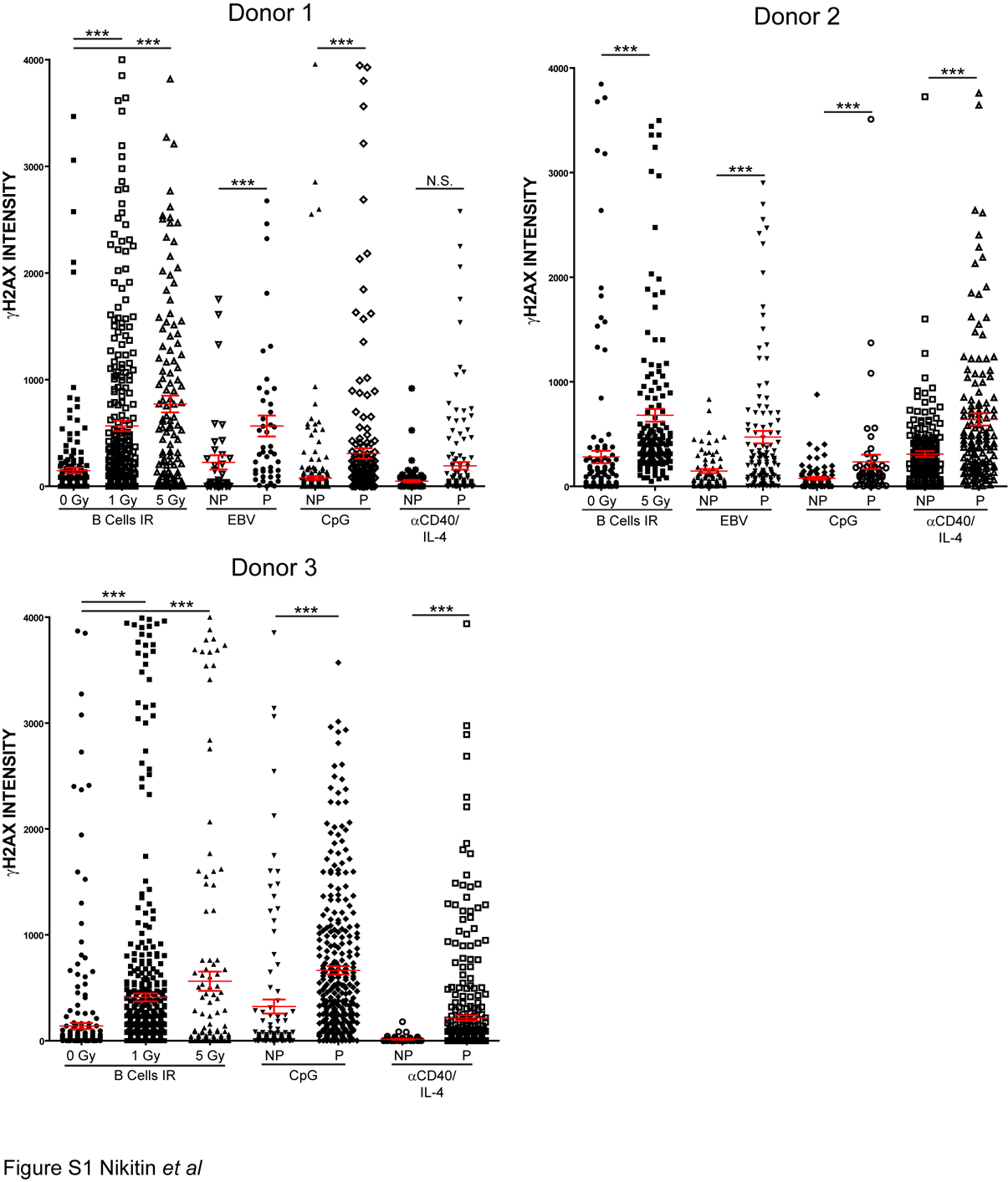

Supplement: Figure S1 — Quantification of γ-H2AX Immuno-Fluorescence intensity. Intensity for γ-H2AX immune-fluorescence staining for three normal human donors shown in Figure 2B is displayed in column format. Samples include B cells, untreated (0), 1 Gy, or 5Gy γ-irradiation, sorted non proliferating (NP) and proliferating (P) cells for EBV, CpG, or αCD40/IL-4 treatments. Mean fluorescence is shown by red bars. Significance was calculated using the non-parametric Mann-Whitney U test. ***, P-value <0.001; N.S., not significant. (TIF) [file pone.0087299.s001.tif]
